# Small vertebrates are key elements in the frugivory networks of a hyperdiverse tropical forest

**DOI:** 10.1038/s41598-020-67326-6

**Published:** 2020-06-29

**Authors:** Daiane C. Carreira, Wesley Dáttilo, Dáfini L. Bruno, Alexandre Reis Percequillo, Katia M. P. M. B. Ferraz, Mauro Galetti

**Affiliations:** 1 Programa Interunidades de Pós Graduação em Ecologia Aplicada, Escola Superior de Agricultura “Luiz de Queiroz”- Universidade de São Paulo (ESALQ-USP), Piracicaba, São Paulo CP 13418-900 Brazil; 2 Fundação Hermínio Ometto - FHO|Uniararas, Araras, São Paulo CP 13607-339 Brazil; 3 Red de Ecoetología, Instituto de Ecología A.C., CP 91070 Xalapa, Veracruz Mexico; 4 Programa de Pós Graduação em Ecologia e Recursos Naturais – Universidade Federal de São Carlos (UFSCar), São Carlos, São Paulo CP 13565-905 Brazil; 5 Departamento de Ciências Biológicas, Escola Superior de Agricultura “Luiz de Queiroz” - Universidade de São Paulo (ESALQ-USP), Piracicaba, São Paulo CP 13418-900 Brazil; 6 Departamento de Ciências Florestais, Escola Superior de Agricultura “Luiz de Queiroz” - Universidade de São Paulo (ESALQ-USP), Piracicaba, São Paulo CP 13418-900 Brazil; 7 Department of Biology, University of Miami, Coral Gables, FL CP 33146 USA; 8 Departamento de Biodiversidade, Universidade Estadual Paulista (UNESP), Rio Claro, São Paulo CP 13506-900 Brazil

**Keywords:** Food webs, Conservation biology

## Abstract

The local, global or functional extinction of species or populations of animals, known as defaunation, can erode important ecological services in tropical forests. Many mutualistic interactions, such as seed dispersal of large seeded plants, can be lost in large continuous forests due to the rarity of large-bodied mammalian frugivores. Most of studies that try to elucidate the effects of defaunation on seed dispersal focused on primates or birds, and we lack a detailed understanding on the interactions between ground-dwelling fauna and fleshy fruits. Using camera traps in forest areas with different degrees of defaunation, we described the organization of frugivory networks involving birds, mammals and plants. We recorded 375 frugivory interactions between 21 frugivores and 150 fruiting trees of 30 species of fleshy fruit plants in six sites in continuous Atlantic forest of Brazil. We found that small frugivores—particularly small rodents and birds—were responsible for 72% of the events of frugivory. Large frugivores, such as tapirs and peccaries, were responsible for less than 21% of frugivory events. Our results indicate that the interactions between flesh fruiting plants and frugivores are dominated by small frugivores, an indication of a functional loss of large frugivores in this endangered biome.

## Introduction

Defaunation processes driven by habitat loss^1^ and poaching^2^ can change interaction patterns and the functional roles that species play within ecological networks^3–5^. Therefore, defaunation is expected to trigger eco-evolutionary processes that propagate across the whole community, reshaping biodiversity and ecosystem functioning^6^. In tropical forests, the loss of large-bodied animals has been shown to reduce functional trait diversity and affect key ecosystem functions, such as herbivory and seed dispersal^7–9^. For example, increases in small mammal densities due to competitive release following local extinction of ungulates^10–12^ have major consequences for seed dispersal^11^ and can reshape the architecture of plant assemblages via cascading effects on seedling recruitment and herbivory^13,14^.

Growing evidence shows that the eco-evolutionary consequences of defaunation for ecological communities are a key but overlooked aspect of the ongoing biodiversity crisis. As ecological interactions represent a pivotal connection between community structure and ecosystem functions^15^, increasing rates of functional extinctions^16^ can disrupt ecosystem services even before species become locally extinct^17,18^. The impacts of functional extinctions of ecological interactions on ecosystem services can be illustrated by the magnitude of reciprocal dependences between topical plants and frugivores: 90% of all plants in tropical forests depend on animals for seed dispersal^19^, and fruits represent 80% of the primary food source for tropical birds and mammals^20^. Given that functional extinctions of ecological interactions currently represent a pervasive process that is changing hyperdiverse tropical ecosystems^16^, it is crucial to understand how novel topological patterns emerging in defaunated species-interaction networks can trigger ecological and evolutionary changes that dynamically reshape the distribution of traits and interactions within anthropogenic communities. This is a key challenge that ecologists shall face in the years to come in order to support innovative, theory-driven strategies of conservation and restoration of resilient and functionally diverse communities^21^.

A first step to understand the eco-evolutionary consequences of defaunation for the long-term dynamics of ecological networks is to use high-quality species-interaction data to describe how anthropogenic extinctions affect the organization of different types of ecological networks. Mutualistic networks, for instance, show recurrent structural patterns, such as nestedness and modularity, that drive ecological and evolutionary dynamics at the community level in different ways^22^. *Nestedness* refers to a structural pattern of interactions formed by one highly interactive core of generalist species that interacts with most species within the network and specialist species that interact mostly with generalists^23^. It arguably promotes ecological stability^22,24–26^ and patterns of trait evolution^22^. *Modularity* refers to species sets that interact more frequently with each other than with the rest of the species in the network^27^. Modular networks imply higher functional diversity^28^, minimize the propagation of perturbations within the network^29^, and can have strong coevolutionary dynamics that shape trait evolution and diversification. In addition, with modular networks it is reasonable to expect that defaunation will reshape species topological roles—i.e., the balance between species connectivity within and between modules^30^. Therefore, the characterization of species topological roles within defaunated assemblages can greatly improve conservation and restoration strategies by allowing us to identify the emerging drivers of community dynamics and design strategies of community manipulation aimed to improve ecosystem functioning^31,32^.

Here, we assessed how vertebrate-plant interaction networks in the forest’s understory are affected by defaunation in the Brazilian Atlantic forest. Specifically, we addressed the following questions: (1) What are the structural frugivory pattern networks on the forest floor involving plants, birds and mammals in the Brazilian Atlantic forest? (2) How does defaunation affect the structure of these networks? (3) How does the relative contribution of species to the organization of the network vary between networks under contrasting degrees of defaunation? We hypothesized that sites subject to higher degrees of defaunation would present contrasting network structures compared to those Atlantic forest sites that face relatively low defaunation pressures. Particularly, we predicted that the sites subject to lower degrees of defaunation pressures hold more species and interactions and show higher degrees of modularity^33–35^. The latter prediction is a logical consequence of our assumption that modularity protects disturbed communities against species loss^35^ and hence species-richer modular communities would be more likely to persist in anthropogenic landscapes. Conversely, networks under higher degrees of defaunation would be more nested and less modular because the extinction of more specialized interactions are expected to be more frequent^33^. Also, the interactions that would persist would be those involving generalist plants and birds, which are more likely to disperse over fragmented landscapes^34^. As defaunation can favor population increases of small mammal species, such as rodents^11^, their contribution to network structure is expected to be higher in those networks facing higher defaunation pressures. By addressing these questions, we seek to advance the current understanding on the consequences of defaunation for the structure of hyperdiverse tropical networks and for the ecological roles played by species within them, a problem that currently represents a major challenge for both theoretical and applied ecologists^30,34,36–38^.

## Material and methods

### Study sites

The Atlantic forest of the coastal and interior parts of Brazil, Paraguay and Argentina once covered 1,500,000 km^2^ from 3 to 30° S^39^. Today only 12% of the forest remains mostly in small fragments highly defaunated of large vertebrates. However, there are two large blocks with more than 10,000 km^2^ that still persist: the Serra do Mar, in eastern Brazil and Misiones, in northeastern Argentina, Paraguay and Brazil.

In this study, we have focused in the Serra do Mar massif, where the largest remaining populations of large mammals are located. Historical and recent hunting pressure, along with habitat loss, have caused local population declines and even extinctions, resulting in a mosaic of mammal biomass. We have chosen six sites along the continuous Serra do Mar massif under contrasting degrees of defaunation to test the effects of defaunation in plant-animal interactions. The areas were selected based on the following criteria: (i) similar vegetation—dense ombrophilous type; (ii) protected by the Brazilian government; (iii) present distinct fauna communities, especially top predators and large frugivores. These study sites are divided in three major regions: (i) Serra do Mar, where is located the Serra do Mar State Park—nucleus of Santa Virginia, with two sampling sites, Itamambuca (SV-I) and Vargem Grande (SV-VG); (ii) Serra de Paranapiacaba, where Carlos Botelho (CB) and Intervales (Int) State Parks are located; (iii) Continental Islands, with two big land-bridge islands, Ilha do Cardoso State Park (IC) and Ilhabela State Park (IB). The two selected islands are considered continental islands; historically, had records of the same large frugivores present in continental areas and currently suffer from intense defaunation due to illegal hunting and deforestation (Supplementary Material S1 online, for details).

### Sampling design

We collected data from Dec-2015 to Dec-2017 using camera traps equipped with infrared triggers (Bushnell Trophy Cam, 8MP) to sample interactions of terrestrial vertebrates and fruiting plants. We used 189 camera traps (SV-I—24, SV-VG—29, CB—35, Int—34, IC—34 and IB—33) and each camera was active on average 60 days (24 h per day). The six sites were sampled during dry and rain seasons and were not sampled simultaneously. We installed each camera at 10–20 cm above the ground and under the projection area of a tree. Chosen trees were common in the area with characteristics of wide and fleshy fruit, attractive to birds and mammals. The minimum distance between camera-armed fruiting trees was 50 m in order to avoid spatial correlation.

We configured cameras to record videos for 30 s, with intervals of thirty seconds between videos, with date-time stamp enabled. For all images obtained, we identified the terrestrial species (and when it was not possible, we identified the genus or family) with the help of experts and specialized literature. We classified the events into two types: 1. Visits—when the animal passed by the camera and did not remove fruits and 2. Frugivory—when there was fruit removal during the passage of the animal by the camera. In this case, each independent record of the animal was considered a frugivory event. We considered an independent record when the individual recorded left the image and did not return during the 30 s of video^9^. We also registered the number of individuals per event.

### Network analysis

We constructed interaction matrixes generating graphical representations of interactions between plants and frugivores for each site, using bipartite graphs^40^. We calculated frequencies and diversity of interactions (Shannon Diversity) between each frugivore and plant species across all areas together and also for each site individually.

We used the following descriptors to characterize the network structure of each area: modularity and nestedness. Modularity means there are groups of frugivore species that strongly interact with a particular set of plant species^30^, while nestedness suggests an asymmetric specialization of the community, in which frugivore species with few interactions ('specialists') preferentially interact with plant species with many interactions ('generalists') and vice versa. This type of nested organization is non-random because it favors the formation of communities more resistant to environmental disturbances, since generalist species form dense sets of interactions.

We calculated weighted modularity (Q) computed by the QuanBiMo algorithm^41^, where Q ranges from 0 (no subgroups) to 1 (totally separated subgroups). For nestedness, we used the WNODF (Weighted Nestedness metric based on Overlap and Decreasing Fill), a nestedness descriptor that varies from zero (non nested) to 100 (perfectly nested)^42^. The significances of both modularity and nestedness were assessed by randomization, using the r2dtable null model based on the Patefield algorithm^43^. This null model uses fixed marginal totals to distribute the interactions and produce a set of networks where all species are randomly associated^23^. We used only weighted nestedness and modularity, mainly because recent studies have shown that binary networks tend to be more sensitive to sampling bias^44^.

We recorded the network roles of species in the modular structure (in each area, separately) by computing the standardized within-module degree (*zi*), which is a measure of the extent to which each species is connected to the other species in its module and the among-module connectivity (*ci*), which describes how evenly distributed are the interactions of a given species across modules. For this, we generated cutoffs of the frequency distribution of *z* and *c* values at the 95% (based on the mean, from lowest to highest values)^45^ and classified species as peripherals (i.e., with a few interactions with other species), connectors (i.e., connects several modules to each other), module hubs (i.e., has several interactions within its module), or network hubs (i.e., the species is a connector and has several interactions in the module)^30^.

Additionally, we also estimated species strength, which determines the relative importance of a frugivore for a particular plant species. For that we calculated the relative frequency with which a frugivore species connects to a plant species divided by the total number of interactions of all frugivore species to that plant species. Before, we considered the sum of interaction strength values of a specific frugivore to all plants^40^. For this analysis we used the bipartite package in the software R^46^.

We tested whether species strength differed between frugivorous groups (i.e., between bird and mammal frugivores, and between rodents and ungulates) within each site (i.e. CB, SV-I, INT, IC, SV-VG and IB) using non-parametric tests (Kruskal and Wilcoxon). In this first case, species were considered as replicates and mammal/birds groups as categories. We also tested whether the strength of species (without grouping the frugivores) differed between areas. Our aim as to test whether any of the areas could have stronger frugivory relations between species than others. In the latter case, we used an Analysis of Variance (ANOVA), where the species were considered replicates and the sites considered as categories.

### Defaunation index

We used the defaunation index *Di* to determine a defaunation gradient between the areas. The *Di* is a quantitative index that can be used to compare ecological communities, through a weighted measure of dissimilarity between the current assemblage of a given location and a reference assemblage from areas with a historical and/or unperturbed state^47^. This index ranges from 0, when there is no difference between the observed and reference assemblages (non-defaunated), to 1, when all species are absent (complete defaunation).

We calculated the frequency (abundance sp.1/total abundance × 100) of species of medium and large mammals recorded in the six areas studied and compared with the reference data based in 113 Neotropical mammalian communities^47^. For each area we have the frequency of 11 species of medium and large mammal species that are consistently detected: *Puma concolor, Panthera onca, Leopardus* sp*., Mazama* sp*., Tayassu pecari, Pecari tajacu, Eira barbara, Tapirus terrestris, Dasyprocta* sp*., Dasypus* sp*.,* and *Nasua nasua*. We excluded species that are not associated with forests, such as *Cerdocyon thous*, and species that are difficult to detect by our method, such as primates and domestic and invasive species. To attribute the importance of the species, we use the same two criteria used by Giacomini and Galetti^47^, that are: D1—species importance value (ω) is indicated by body size elevated to the power of ¾^48^, which is a good predictor of the vulnerability of the species^49,50^ and D2—all species have the same importance (ω = 1—^47^). Using the formula proposed by Giacomini and Galetti^47^, we calculated the data through a routine in R.

To test whether the rate of defaunation varied according to species strength of rodents and ungulates, we performed linear regressions. All analyses were performed using R (v. 3.5.3; R Core Team 2019).

## Results

### Sampling effort

We had an average sampling effort of 60 trap-days per camera, totalizing 140,040 sampling hours, in 150 fruiting trees of 30 species across all six sites. We identified 104 vertebrate species, being 44 (42.4%) mammal taxa (33 identified on species level, six at the genus level and five at the family/group level; these are mainly small mammals, that could not be identified on lower taxonomic levels with the videos available) and 60 (57.6%) bird species (eight at the genus level and two at the family level) (Supplementary Material S2 online, for details).

Regarding the 44 species of mammals, we detected 21 (47.7%) frugivores, 11 (25%) carnivores, three (6.8%) insectivores, two (4.5%) omnivores and two (4.5%) herbivores. From the 60 bird species, we detected a total of 21 (35%) omnivores, 17 (28.3%) insectivores, 16 (26.6%) frugivores and four (6.6%) carnivores.

**Figure 1:**
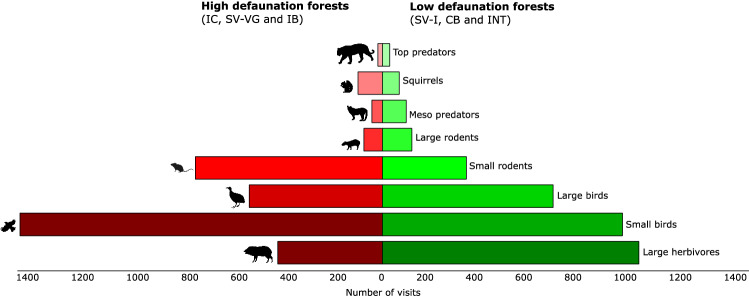
Visitation rates on fruiting trees by birds and mammals in areas with low defaunation (SV-I, CB and INT) and high defaunation (IC, SV-VG and IB) in the Atlantic forest, Brazil. States Parks: SV-I: Itamambuca, CB: Carlos Botelho; INT: Intervales, IC. Ilha do Cardoso, SV-VG: Vargem Grande and IB: Ilhabela. Animal silhouettes are from PhyloPic (URL: https://phylopic.org/) under a CC BY open access license.

We established the defaunation gradient using the index of defaunation of each sampled area. We classified SV-I (0.233), CB (0.312) and INT (0.321) as low defaunation areas and IC (0.686), SV-VG (0.735) and IB 0.953, as high defaunation areas (Supplementary Material S3 online, for details). In low defaunation areas large herbivores had the highest visitation number (1.026 visits), while small birds (1.442 visits) and small rodents (744 visits) had the highest rates within high defaunation areas (Fig. 1).

### The structure of fruit-frugivore networks

We recorded 6,820 interactions, an average of 1,136 interactions per site between mammals and birds and 150 trees of 30 species. Of these interactions, 76 (74%) animal species were identified only as visitors (29 mammal and 47 bird species) while 27 (26%) species removed fruits (15 mammal and 12 bird species). The species that removed fruits performed a total of 375 (5.5%) interactions, being 238 (63.5%) interactions in low defaunation areas and 137 (36.5%) interactions in high defaunation areas. Frugivory interactions varied according to sites, and the areas that presented the most interactions were Itamambuca (33%), Carlos Botelho (25%), Vargem Grande (14%), Ilha do Cardoso (12%), Ilhabela (10%) and Intervales (6%).

**Figure 2:**
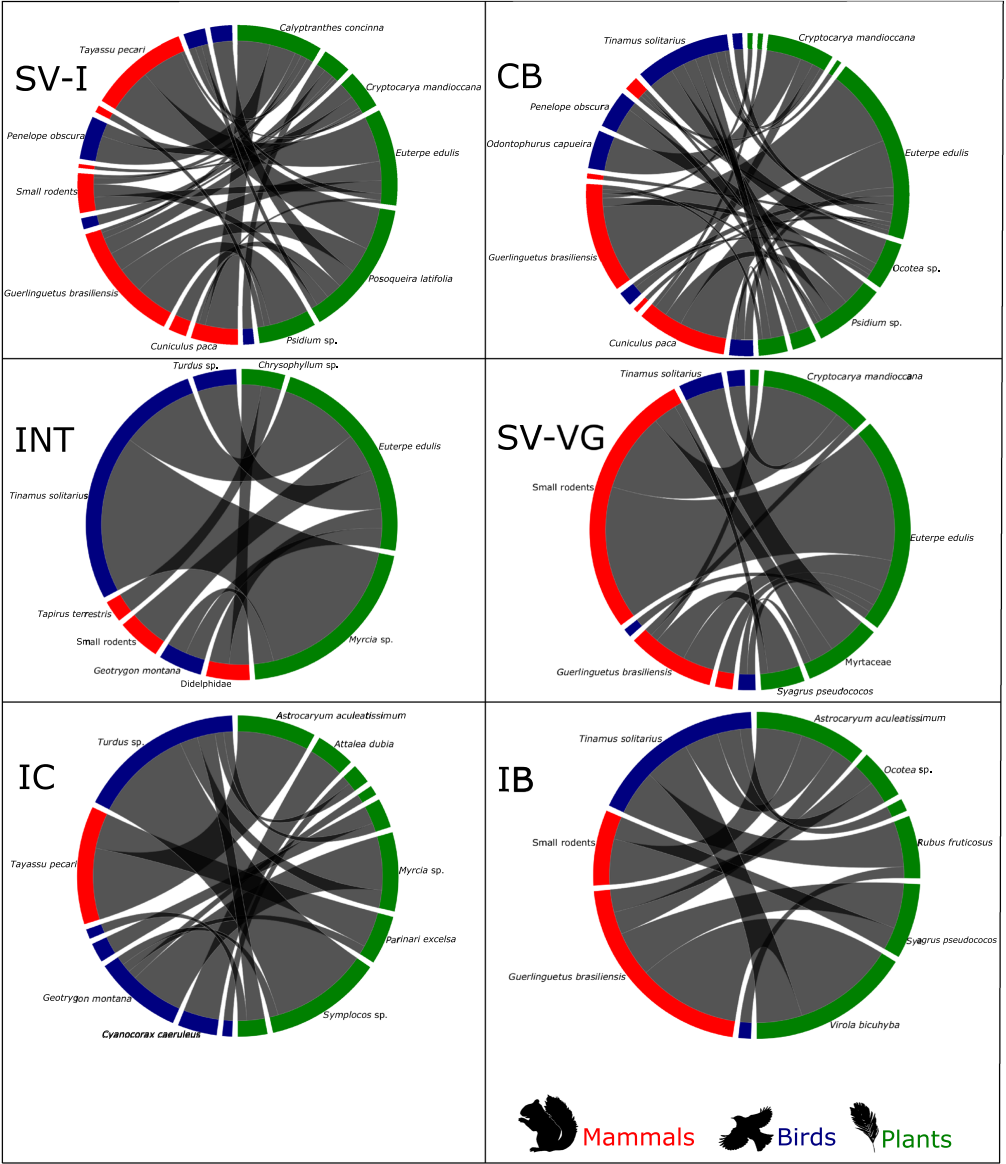
The plant frugivore network in the Atlantic forest, Brazil connected by interactions (gray lines) between plants and frugivores (mammals and birds). In both classes, green indicates plant species, red indicates mammals and blue indicates birds. The largest the size of the colored symbol more interactions that species have. States Parks: SV-I: Itamambuca, CB: Carlos Botelho; INT: Intervales, IC. Ilha do Cardoso, SV-VG: Vargem Grande and IB: Ilhabela. Animal and plant silhouettes are from PhyloPic (URL: https://phylopic.org/) under a CC BY open access license.

We found variance in quantity of fruit removal by species in each area analyzed. In Itamambuca, Carlos Botelho and Ilhabela, were the squirrels (*Guerlinguetus brasiliensis)* with 34, 23 and 17 frugivory events, respectively; in Vargem Grande, were small rodents, with 31 events; in Ilha do Cardoso, were thrush *(Turdus* sp.*)* with 17 events; and finally, in Intervales, were the solitary Tinamoy *(Tinamus solitarius)* with 12 events (Fig. 2).

The diversity of interactions considering all areas (Shannon diversity) was 4.137 and varied little between areas. Intervales, had the smallest Shannon diversity (1.85) and Itamambuca he greatest with (2.99). All networks presented significant modularity (Z-score > 2) (Supplementary Material S4 online, for details) and no network presented a nested pattern of species interactions (Table 1).

**Table 1:** Descriptors of interaction networks between plants and frugivores (birds and mammals) in six areas of the Atlantic forest, in southeastern Brazil.

	All areas	SV_-I	CB	INT	IC	SV_-VG	IB
No. of interactions	375	124	93	21	46	53	38
Diversity of interactions	4.137	2.99	2.95	1.85	2.32	2.15	2.30
Modularity (Q)	0.39	0.41	0.39	0.32	0.61	0.26	0.29
Z-score (modularity)	16.96	11.62	7.88	2.20	9.68	3.27	2.60
Nestedness (WNODF)	25.09	18.33	37.63	11.11	3.94	35.48	32.14
Z-score (nestedness)	− 2.71	− 5,13	− 0.90	− 1.87	− 3.75	− 1.44	− 0.93

Interactions, number of interactions; Diversity of interactions, Shannon diversity; Mod, Modularity; NODF, Nestedness. States Parks: SV-I: Itamambuca, CB: Carlos Botelho; INT: Intervales, IC. Ilha do Cardoso, SV-VG: Vargem Grande and IB: Ilhabela.

**Figure 3:**
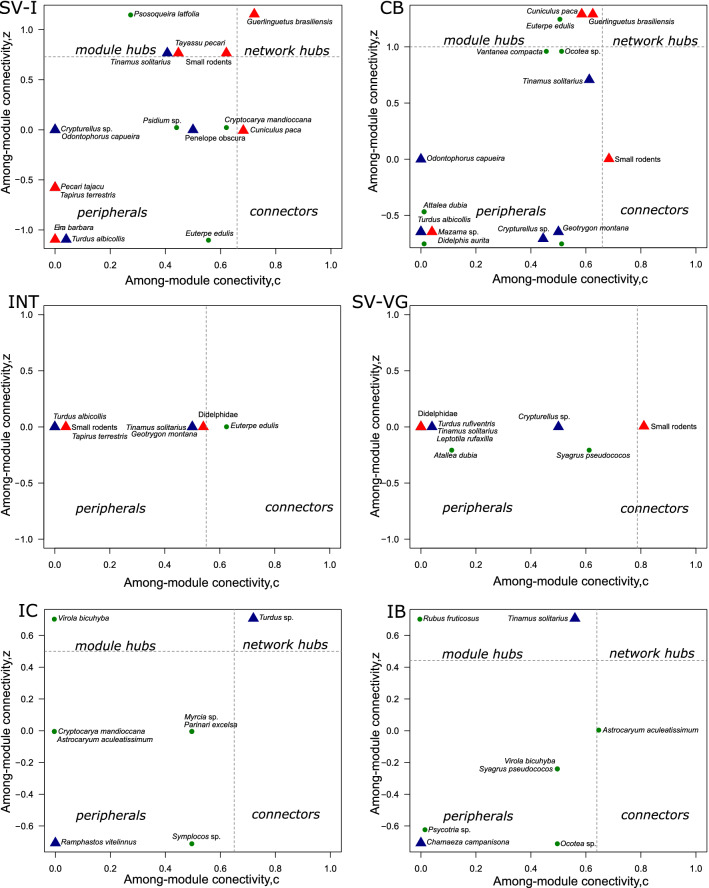
Network roles of different species of frugivores mammals and birds and plants, in the multi-interaction network of the Atlantic forest, in southeastern Brazil. The variable within module degree (z) describes the standardized number of interactions of a species compared with other species in it's module. The variable among module connectivity (c) describes the distribution of interaction of a given species across partner species in different modules. States Parks: SV-I: Itamambuca, CB: Carlos Botelho; INT: Intervales, IC. Ilha do Cardoso, SV-VG: Vargem Grande and IB: Ilhabela.

When considering the multi-interaction network, we identified that only *Guerlinguetus brasiliensis* acts like a “network hub” in Itamambuca, and *Turdus* sp. in Ilha do Cardoso. The other areas did not have “network hubs”. Connector species were *Cuniculus paca* in Itamambuca, for Carlos Botelho and Vargem Grande small rodents. The module species were *Tayassu pecari*, *Tinamus solitarius* and small rodents in Itamambuca; *Cuniculus paca* and *Guerlinguetus brasiliensis* in Carlos Botelho, and *Tinamus solitarius* in Ilhabela. Vargem Grande did not present module species, Ilhabela area didn't present connectors species and in Ilha do Cardoso, all species were peripherals. Most plant and animal species were peripherals in all areas studied (Fig. 3).

### Species and important groups in the networks

Species strength of frugivores ranged from 0.03 to 3.09 (Supplementary Material S5 online, for details), but did not differ between areas (p = 0.14). However, some species presented higher values of interaction strength than others (> 1.00). For instance, *Tinamus solitarius*—a large frugivore bird, was the species with more force in Carlos Botelho (3.09), Ilhabela (2.9) and Intervales (1.29); *Guerlinguetus brasiliensis*—a small rodent, in Vargem Grande (2.04) and Itamambuca (1.84); and *Turdus* sp. a small and generalist bird, in Ilha do Cardoso (2.67). C*uniculus paca*—a medium frugivore—also appeared with great interaction force in Carlos Botelho (1.76). Small rodents were strong in Vargem Grande (1.98) and *Geotrygon montana*—a common dove and *Tayassu pecari*—a large frugivore mammal—in Ilha do Cardoso (2.03 and 1.08, respectively).

Plant species strength ranged from 0.04 to 5.86, and the species with greater strength were *Euterpe edulis* in Carlos Botelho (5.86), Vargem Grande (3.45) and Intervales (3.33); *Casearia sylvestris* in Itamambuca (2.12); *Rubus fruticosus* in Ilhabela (1.3), and *Virola bicuhyba* in Ilha do Cardoso (1.12).

We found that in Ilha do Cardoso, the bird community, though not significant (p = 0.55), had a greater species strength when compared to that from other areas (7.20 species strength) Itamambuca, had a greater mammal species strength when compared to that from other areas (4.16 species strength); however, this difference, as with the birds, was not significant either (p = 0.42). When we compared the species strength between birds and mammals considering all areas, we didn't find significant differences as well (p = 0.93).

We tested the hypothesis that defaunation could be inversely related to ungulate species strength (areas with less defaunation would have higher ungulate species strength) and did not find significant correlation (p = 0.54, R^2^ = 0.09). In addition, when we tested if areas with more defaunation would have higher rodent species strength, the importance of rodents in networks increased as areas became more defaunated (p = 0.03, R^2^ = 0.70).

## Discussion

### Active frugivores in the Atlantic Forest understory

We found that despite bird and mammal communities being distinct in composition, the frugivory rate was very low in all areas. Some sites as Carlos Botelho and Itamambuca, even though still presenting large mammals (jaguar and tapirs), revealed that frugivory interactions are not occurring or were not detected. Importantly, our study evaluated only frugivory interactions that occur on the forest floor. There are several other frugivore agents acting on the forest canopy, such as primates, bats and large birds (toucans and toucanets), that were not covered in this study. It is important to highlight that not detecting frugivory by large mammals (peccaries, tapirs and mazamas, for example) may be a warning sign for the loss of interactions.

Frugivores that removed the most fruits varied according to the study areas, but in general were small (300 gr), except for one tinamid, which can weigh up to 1.5 kg. In SV-I, CB and IB, were squirrels that most removed fruits under fruiting trees. The registered species is a generalist squirrel (*Guerlinguetus brasiliensis*). It is widely distributed in the Atlantic forest^51^. This species acts primarily as seed predator^11^ and assists in the control of seedling density in tropical forests^52,53^. Despite its primary role as seed predator, as well as others scatter-hoarding tropical rodents, squirrels also bury mature seeds acting as secondary seed dispersers^54^.

Small rodent mammals also played an important role in fruit removal in SV-VG. Several species were recorded removing fruit including some marsupials. It is well known that most rodents are effective seed predators that can compromise seedling recruitment; however, it will depend on a number of post-dispersal factors such as the microsites the seed will be deposited on, competition with other species present, and even the size of the seed which will determine the extent of predation^55,56^.

Birds had high fruit removal in IC and INT. In IC, thrush (*Turdus* sp.) were the most effective in removing. Thrushes can be important seed dispersers and contribute to plant regeneration, especially in degraded areas where they are quite common. One study showed that thrushes can track fruit in a forest, especially in years with low fruiting abundance, and they would tend to disperse seeds in areas with poor forest cover^57^.

Solitary Tinamou (*Tinamus solitarius*)—which has high fruit removal in INT, is a tinamiforme endemic to the Atlantic Forest^58^, near threatened species (IUCN), which is present in areas with high degrees of defaunation^11^. This bird is considered cinegetic and, in the forest continuum where the Intervales Park is inserted, it is commonly consumed by people who illegally collect hearts of palm^59^—*Euterpe edulis*, an endangered species of endemic palm of the Atlantic Forest. Little is known about fruit consumption by this bird species and only one study mentions that it can be a predator of hard seeds, through the digestion process, of *Syagrus rommanzofiana*—a palm^60^.

### Structure of networks and species role

In our study, we found that all networks are modular (even networks with low species diversity) and no network was significantly nested when compared with the neutral patterns of species interactions (null models). Modularity takes into account the affinity of the links, and to be modular allows the networks to be more stable in face of disturbances. This is because they present a series of animal species interacting with the same plant, and in case of losing one frugivore species, others would continue to interact with that plant. Hence, disturbances would be felt more slowly in modular networks than in non-modular networks^30^. In addition, modular networks may represent a potential for coevolution between plants and animals^61^.

In the analysis of the structural composition of networks, very connected species are fundamental for the organization of the networks^30^. These species may play central roles as “*hubs*” when they have a disproportionally large number of interactions, as "*connectors*" when a species binds different modules of the network, or as both^62^. In this study, we found that network hubs are present only in two areas (SV-I and IC). In SV-I is a squirrel (*G. brasiliensis*) and in IC, thrushes (*Turdus* sp.). The species that act as connectors are identify in three areas: SV-I—*Cuniculus paca* (a large rodent), and CB and SV-VG—small rodents. These results support the initial data that small mammal species are largely responsible for the removal of fruits in the forest understory. These species, although generalists, are important in structuring the networks, as they are connected to other species (plants and frugivores). In simulation studies, hubs species are responsible for the robustness of the networks, thus, the functional extinction of one of them could lead to a rapid collapse of the network^35^.

Some species presented low importance in frugivory networks acting as module hubs (i.e. *T. solitarius, Tayasuu pecari* and small rodents in SV-I; *C. paca* and *G. brasiliensis* in CB, and *T. solitarius* in IB) which have many interactions but only within their own modules (interacting with the same plant species), opposing to network hubs that interact with different species at a very high rates. Peripheral species present low rates of frugivory interactions. Most species of large mammals (*Tapirus terrestris, Mazama* sp. and *Pecari tajacu Tayassu pecari*—this last, except in SV-I, which appears as module hub) presented low frugivory rates in all areas, suggesting that the rate of defaunation does not affect frugivory rates.

The effects of losing certain species will depend on their role within the frugivory networks. Extinction of module species can cause fragmentation of interactions within its own module redistributing new interactions between the other species of the module, but its impact will be minimal on other network modules^63^. Nonetheless, the loss of connector or hub species can cause a rupture in interactions, fragmenting the network into several isolated modules^30^. In addition to affecting directly or indirectly other interactions within the network^64^, it may even promote secondary extinctions^65^ if plant species (for instance) lose their potential dispersers.

All species contributing most to the organization of networks (higher species strength values)—*T. solitarius*, *G. brasiliensis*, *Turdus* sp., *C. paca*, *Geotrygon montana*, *T. pecari* and small rodents are considered seed predators^11^. Only *C. paca* is considered a potential seed disperser since, after eating the pulp, it discards the endocarps or might create endocarp or agglomerates on the ground^66^. Seed predation is also considered an important mechanism that assists in the control of seedling density in tropical forests^52,53^. The variation in species interaction patterns can be explained by some factors such as the variation in the abundance of resources and consequently the change in diet by some animals and the morphology of the species. These effects could be noticed in the structure of the networks and changes of the roles of the species in the interactions with the plants.^67^.

### Frugivory and defaunation

When we look at the groups that participate in the frugivory interactions in the networks, we see that large mammals contribute little to the interactions and there is no relationship between defaunation and frugivory rate for these animals. On the other hand, small rodents are benefited by the defaunation, increasing its importance in the frugivory interactions. Our results agree with other authors who verified an increase in the abundance and diversity of small mammals in defaunated areas^9,10,68,69^. In yet another study, small rodents were responsible for 98% of the predated seeds in defaunated areas, while in non-defaunated areas they responded for 63% of seed predation^11^. Thus, defaunation could favor this group in terms of numbers, which in turn would compensate the lack of seed predation by peccaries and tapirs, for example^11^. Some factors should explain the permanence and abundance of these small rodents in areas more defaunated, such as their high reproductive rate and the fact that they are less vulnerable to human disturbances^10,70,71^, when compared to large mammals, affected by poaching and habitat fragmentation^72,73^.

Defaunation in the Atlantic forest does not seem to be directly related to frugivory processes. Even in areas where large mammals remain present, they practice little or no frugivory activity. This functional change in networks of interactions in the Atlantic forest, where large mammals cease to act as frugivores, removing fruits may compromise secondary processes to frugivory, such as seed dispersal. Changing the community of frugivores that effectively remove fruits may lead to changes in the composition of forest species^5^, affecting its structure and dynamics of several plants^74^. However, we must also consider the role of primates and large birds that feed on the canopy and that were not contemplated by this methodology. What we can affirm is that in the forest floor the frugivory has been carried out mainly by birds and rodents, usually generalists and seed predators. Also, in areas with high levels of defaunation, rodents increase their importance in interactions, visiting and removing many fruits of native species.

## Considerations

The loss of interactions can precede the loss of the species^18,75^, functionally important for the maintenance of the ecosystem. In this sense, we point out two ways that could be explored in order to maintain the conservation of the system's functionality.

The first is to maintain viable minimum communities of threatened species and prevent the extinction of new species. Authors have especially defended the permanence of keystone species in tropical forests. These species would be responsible for maintaining the community structure and ecosystem functioning^76,77^, like herbivores that keep plant abundance below a critical threshold^18^, large predators, fundamental in the regulation of large prey, including ungulates^78^ and large specialized frugivores, responsible for long-distance seed dispersal, decreasing the chances of seedling predation^49,53,79,80^.

A second way would be to identify the threshold of change in species behavior. In this case, we suggest the identification of zone-type thresholds, based on the models proposed by Muradian^81^ and Huggett^82^. These threshold models show a gradual shift from one state to another (in our example, the decline of frugivory interactions), rather than an abrupt change. At this threshold, you do not consider changes in the independent variable (e.g. forest loss) or impact on the dependent variable (e.g. species richness). One of the limitations of this model is the difficulty of establishing a threshold on the interactions, because the environments can behave differently according to the region they are inserted, or the degrees of threat they suffer^82^.

We acknowledge that identifying this threshold is not easy, since it is necessary to detect the change that occurs for species to cease playing their ecological role, such as frugivory and seed dispersal. Therefore, we suggest a vast cataloging of the abundances of key species to frugivory and the frugivory interactions they actually promote in their regions, and then applying a theoretical model that can detect the transition zone in the frugivory behavior of that species—at what moment in population, the species stop practicing frugivory or decrease its performance. From this identification, the conservation actions could be directed.

We point out here also, some considerations important to guide future studies. we suggest extending sampling effort to other strata of the forest in order to identify all active frugivores and characterize the potential of frugivores as effective seed dispersers, through laboratory and field experiments. Finally, we point to the need to diagnose the thresholds of effectiveness of mammals in frugivory in tropical forests with a great history of human disturbances^83^ and overhunting^84^, such as the Atlantic forest.

## Supplementary information

Supplementary file1
