# Supplementary material for: Small vertebrates are key elements in the frugivory networks of a hyperdiverse tropical forest
Source: Sci Rep. 2020 Jun 29;10:10594. doi: 10.1038/s41598-020-67326-6 (PMC7324603; doi:10.1038/s41598-020-67326-6)
Supplement: Supplementary file 1 — Supplementary file1 [file 41598_2020_67326_MOESM1_ESM.pdf]

Small vertebrates are key elements in the frugivory networks of a hyperdiverse tropical forest

Daiane C. Carreira, Wesley Dáttilo, Dáfini L. Bruno, Alexandre Percequillo, Katia M.P.M.B Ferraz and Mauro Galetti

Electronic Supplemental Material 1

Climatic and vegetative characteristics of the study sites, located in Serra do Mar - Serra do Mar State Park – nucleus of Santa Virginia, Itamambuca and Vargem Grande; Serra de Paranapiacaba - Carlos Botelho State Park and Intervales and continental islands - Ilha do Cardoso State Park and Ilhabela State Park, in Brazil.

Site	Geographic coordinate	Area size (ha)	Vegetal cover	Annual temperature	Annual rainfall	Reference
Nucleus of Santa Virginia	23º25'S to 44º50'W	315.000	Atlantic ombrophiles dense forest (high and low lands), and secondary forest.	21ºC	2.200 mm	São Paulo 2006
Carlos Botelho State Park	24º 06' S and 47º 47'	37.794	Dense ombrophiles forest (forest primary in step terrains, and by secondary forests in flat areas).	17°C to 22°C	1.700 to 2.400 mm	São Paulo 2008
Intervales State Park	24º15'S, 48º10'W	41.704	Dense ombrophiles forest (forest primary in step terrains, and by secondary forests in flat areas).	21°C	1.600 to 1.700	São Paulo 2008b
Ilha do Cardoso State Park	48º05'W and 25º03'S	15.100	Many types of ecosystems beaches, estuary, rivers, restinga forests, tropical rainforest of	21°C	2.300 mm	São Paulo 2001

			the coastal plain and mangroves.			
Ilhabela State Park	23°57' S and 45°27' O	34.754	The natural areas were historically occupied by sugar cane and small artisanal industries, only in the twentieth century did activities cease and the secondary forests covered much of the perimeter of the island (about 84%). It presents vegetation types of dense ombrophiles lowland and submontane forest, dense montane ombrophiles forest and mountain refuges	22ºC	1.500 mm	São Paulo 2015

Electronic Supplemental Material 2

Abundance of visits (V) and frugivory (F) by birds and mammals in the Atlantic forest, São Paulo, Brazil.

				Total		CB		SV-I		SV-VG		IC		INT		IB	
**Species/Taxa**	**Category**	**Diet**	**IUCN**	**V**	**F**	**V**	**F**	**V**	**F**	**V**	**F**	**V**	**F**	**V**	**F**	**V**	**F**
Tayassu pecari	mammal	very large frugivore	VU	1258	40	51	0	766	28	3	0	438	12	0	0	0	0
Tinamus solitarius	bird	large frugivore	NT	525	56	173	19	30	6	38	5	21	0	113	12	150	14
Turdus albicollis	bird	medium frugivore	LC	491	26	82	2	68	6	42	0	232	16	22	2	45	0
Geotrygon montana	bird	medium frugivore	LC	384	14	24	3	5	0	8	0	294	9	18	2	35	0
Odontophorus capueira	bird	medium frugivore	LC	382	13	124	8	68	3	122	0	35	2	28	0	5	0
Turdus rufiventris	bird	medium frugivore	LC	360	2	4	0	241	0	102	2	7	0	5	0	1	0
Small rodents**	mammal		-	269	12	21	0	11	0	122	10	6	0	30	1	79	1
Chamaeza sp.	bird	omnivore	-	204	0	119	0	13	0	31	0	3	0	17	0	21	0
Crypturellus obsoletus	bird	medium frugivore	LC	191	5	34	3	18	1	108	0	9	1	22	0	0	0
Cuniculus paca	mammal	large frugivore	LC	190	34	69	21	31	13	33	0	0	0	17	0	40	0
Guerlinguetus brasiliensis	mammal	small frugivore	LC	164	83	37	23	27	33	34	10	0	0	3	0	63	17
Formicarius colma	bird	insectivore	LC	156	0	0	0	0	0	1	0	154	0	1	0	0	0
Tapirus terrestris	mammal	very large frugivore	VU	113	3	32	0	48	2	3	0	0	0	30	1	0	0
Sigmodontinae	mammal		-	107	13	6	2	14	0	71	10	1	0	5	0	10	1
Trinomys iheringi	mammal	small frugivore	-	101	15	16	0	0	0	33	11	0	0	0	0	52	4
Grallaria varia	bird	omnivore	LC	87	0	1	0	54	0	28	0	0	0	4	0	0	0
Didelphis aurita	mammal	medium frugivore	LC	80	1	6	1	8	0	8	0	2	0	26	0	30	0
Pyriglena leucoptera	bird	insectivore	LC	73	0	12	0	7	0	38	0	11	0	1	0	4	0
Chamaeza meruloides	bird	omnivore	LC	69	0	37	0	3	0	19	0	6	0	4	0	0	0

Species/Taxa	Category	Diet	IUCN	Total		CB		SV-I		SV-VG		IC		INT		IB	
				V	F	V	F	V	F	V	F	V	F	V	F	V	F
Leptotila rufaxilla	bird	medium frugivore	LC	67	1	4	0	32	0	8	1	1	0	2	0	20	0
Pecari tajacu	mammal	very large frugivore	LC	67	1	2	0	56	1	2	0	0	0	7	0	0	0
Leptotila verreauxi	bird	omnivore	LC	56	0	2	0	10	0	5	0	1	0	8	0	30	0
Leptotila sp.	bird	omnivore	-	52	0	7	0	16	0	10	0	2	0	1	0	16	0
Conopophaga lineata	bird	insectivore	LC	51	0	1	0	2	0	38	0	0	0	1	0	9	0
Didelphidae	mammal	--	-	50	4	0	0	1	0	13	2	1	0	29	2	6	0
Puma concolor	mammal	carnivore	LC	46	0	6	0	11	0	9	0	9	0	11	0	0	0
Rodentia (subfamília Sigmodontinae e família Echimyidae)	mammal	--	-	45	8	9	0	8	4	21	3	5	0	0	1	2	0
Crypturellus sp.	bird	medium frugivore	-	45	6	5	2	7	2	30	2	1	0	2	0	0	0
Mazama sp.	mammal	very large frugivore	-	45	1	18	1	0	0	0	0	11	0	16	0	0	0
Sclerurus scansor	bird	insectivore	LC	44	0	2	0	4	0	22	0	7	0	4	0	5	0
Leopardus pardalis	mammal	carnivore	LC	43	0	10	0	14	0	4	0	6	0	8	0	1	0
Philander frenatus	mammal	small frugivore	LC	41	0	24	0	4	0	2	0	0	0	0	0	11	0
Chamaeza campanisona	bird	small frugivore	LC	41	1	16	0	1	0	3	0	0	0	1	0	20	1
Myrmoderus squamosus	bird	insectivore	LC	41	0	8	0	4	0	25	0	2	0	0	0	2	0
Penelope obscura	bird	large frugivore	LC	41	20	27	8	6	12	3	0	4	0	1	0	0	0
Dasypus novemcinctus	mammal	omnivore	LC	38	0	1	0	1	0	1	0	2	0	0	0	33	0
Nasua nasua	mammal	medium frugivore	LC	35	0	30	0	5	0	0	0	0	0	0	0	0	0
Marmosops sp.	mammal	small frugivore	-	30	0	9	0	8	0	13	0	0	0	0	0	0	0
Myiothlypis rivularis	bird	insectivore	LC	29	0	0	0	5	0	19	0	5	0	0	0	0	0

				Total		CB		SV-I		SV-VG		IC		INT		IB	
**Species/Taxa**	**Category**	**Diet**	**IUCN**	**V**	**F**	**V**	**F**	**V**	**F**	**V**	**F**	**V**	**F**	**V**	**F**	**V**	**F**
Turdus sp.	bird	omnivore	-	26	0	2	0	17	0	4	0	2	0	1	0	0	0
Canis lupus familiaris	mammal	carnivore	LC*	25	0	2	0	3	0	4	0	7	0	1	0	8	0
Metachirus nudicaudatus	mammal	small frugivore	LC	22	0	4	0	1	0	7	0	8	0	1	0	1	0
Cerdocyon thous	mammal	carnivore	LC	20	0	1	0	0	0	4	0	13	0	2	0	0	0
Turdus flavipes	bird	small frugivore	LC	20	1	0	0	0	0	0	0	15	1	4	0	1	0
Eira barbara	mammal	medium frugivore	LC	17	5	4	0	6	5	3	0	0	0	4	0	0	0
Aramides saracura	bird	omnivore	LC	17	0	6	0	2	0	8	0	0	0	1	0	0	0
Caracara plancus	bird	omnivore	LC	17	0	0	0	17	0	0	0	0	0	0	0	0	0
Baryphthengus ruficapillus	bird	omnivore	LC	16	0	5	0	1	0	3	0	1	0	6	0	0	0
Batara cinerea	bird	omnivore	LC	16	0	6	0	0	0	10	0	0	0	0	0	0	0
Oligoryzomys nigripes	mammal	small frugivore	LC	15	5	7	1	8	4	0	0	0	0	0	0	0	0
Aramides sp.	bird	omnivore	-	12	0	0	0	0	0	11	0	0	0	1	0	0	0
Myiothlypis sp.	bird	insectivore	-	12	0	0	0	4	0	6	0	2	0	0	0	0	0
Schiffornis virescens	bird	omnivore	LC	12	0	2	0	1	0	3	0	5	0	0	0	1	0
Conopophaga melanops	bird	insectivore	LC	11	0	2	0	0	0	2	0	0	0	4	0	3	0
Habia rubica	bird	omnivore	LC	10	0	7		1	0	0	0	2	0	0	0	0	0
Dasypus sp.	mammal	omnivore	-	9	0	0	0	0	0	0	0	0	0	1	0	8	0
Aburria jacutinga	bird	large frugivore	EN	9	0	1	0	0	0	0	0	0	0	2	0	6	0
Dendrocolaptidae	bird	--		8	0	0	0	2	0	0	0	6	0	0	0	0	0
Leopardus guttulus	mammal	carnivore	VU	7	0	0	0	2	0	5	0	0	0	0	0	0	0
Marsupialia	mammal	--	-	7	0	2	0	1	0	4	0	0	0	0	0	0	0
Trichothraupis melanops	bird	omnivore	LC	7	0	0	0	0	0	0	0	7	0	0	0	0	0

				Total		CB		SV-I		SV-VG		IC		INT		IB	
**Species/Taxa**	**Category**	**Diet**	**IUCN**	**V**	**F**	**V**	**F**	**V**	**F**	**V**	**F**	**V**	**F**	**V**	**F**	**V**	**F**
Leopardus wiedii	mammal	carnivore	NT	6	0	3	0	2	0	1	0	0	0	0	0	0	0
Leopardus sp.	mammal	carnivore	-	4	0	0	0	0	0	1	0	0	0	2	0	1	0
Myrmecophaga tridactyla	mammal	insectivore	VU	4	0	0	0	0	0	0	0	0	0	4	0	0	0
Tamandua tetradactyla	mammal	insectivore	LC	4	0	1	0	0	0	0	0	1	0	1	0	1	0
Lepus capensis	mammal	herbivore	LC	3	0	3	0	0	0	0	0	0	0	0	0	0	0
Puma yagouaroundi	mammal	carnivore	LC	3	0	0	0	0	0	2	0	1	0	0	0	0	0
Sapajus apella	mammal	medium frugivore	LC	3	1	2	0	0	0	0	0	0	0	0	0	1	1
Sylvilagus brasiliensis	mammal	herbivore	LC	3	0	0	0	3	0	0	0	0	0	0	0	0	0
Automolus leucophthalmus	bird	insectivore	LC	3	0	0	0	0	0	0	0	3	0	0	0	0	0
Drymophila squamata	bird	insectivore	LC	3	0	0	0	0	0	0	0	3	0	0	0	0	0
Merulaxis ater	bird	insectivore	NT	3	0	0	0	0	0	2	0	0	0	0	0	1	0
Tachyphonus coronatus	bird	omnivore	LC	3	0	2	0	0	0	0	0	0	0	0	0	1	0
Cabassous cf. *tatouay*	mammal	insectivore	LC	2	0	0	0	0	0	0	0	1	0	0	0	1	0
Monodelphis cf. *americana*	mammal	small frugivore	LC	2	0	1	0	1	0	0	0	0	0	0	0	0	0
Procyon cancrivorus	mammal	very large frugivore	LC	2	0	0	0	0	0	0	0	0	0	2	0	0	0
Falco femoralis	bird	carnivore	LC	2	0	0	0	2	0	0	0	0	0	0	0	0	0
Strigidae	bird	--	-	2	0	0	0	0	0	1	0	1	0	0	0	0	0
Dasyprocta leporina	mammal	medium frugivore	LC	1	0	0	0	0	0	1	0	0	0	0	0	0	0
Didelphis sp.	mammal	medium frugivore	-	1	0	0	0	0	0	0	0	0	0	0	0	1	0
Felis silvestris catus	mammal	carnivore	LC*	1	0	0	0	0	0	0	0	0	0	0	0	1	0
Lontra longicaudis	mammal	carnivore	NT	1	0	0	0	0	0	1	0	0	0	0	0	0	0
Marmosops cf. *incanus*	mammal	small frugivore	LC	1	0	0	0	0	0	0	0	0	0	1	0	0	0

				Total		CB		SV-I		SV-VG		IC		INT		IB	
**Species/Taxa**	**Category**	**Diet**	**IUCN**	**V**	**F**	**V**	**F**	**V**	**F**	**V**	**F**	**V**	**F**	**V**	**F**	**V**	**F**
Marmosa (Micoureus)	mammal	small frugivore	-	1	0	1	0	0	0	0	0	0	0	0	0	0	0
Panthera onca	mammal	carnivore	NT	1	0	0	0	0	0	0	0	0	0	1	0	0	0
Amadonastur lacernulatus	bird	carnivore	VU	1	0	0	0	0	0	0	0	1	0	0	0	0	0
Celeus flavescens	bird	omnivore	LC	1	0	0	0	0	0	0	0	0	0	0	0	1	0
Colaptes melanochloros	bird	omnivore	LC	1	0	0	0	0	0	0	0	0	0	1	0	0	0
Crypturellus tataupa	bird	omnivore	LC	1	0	0	0	0	0	1	0	0	0	0	0	0	0
Cyanocorax caeruleus	bird	medium frugivore	NT	1	4	0	0	0	0	0	0	1	4	0	0	0	0
Hypoedaleus guttatus	bird	insectivore	LC	1	0	0	0	0	0	0	0	1	0	0	0	0	0
Laterallus sp.	bird	omnivore	-	1	0	0	0	0	0	0	0	1	0	0	0	0	0
Lochmias nematura	bird	insectivore	LC	1	0	0	0	0	0	1	0	0	0	0	0	0	0
Micrastur semitorquatus	bird	carnivore	LC	1	0	0	0	0	0	1	0	0	0	0	0	0	0
Myiothlypis leucoblephara	bird	insectivore	LC	1	0	0	0	1	0	0	0	0	0	0	0	0	0
Penelope sp.	bird	large frugivore	-	1	0	0	0	0	0	0	0	0	0	1	0	0	0
Philydor rufum	bird	insectivore	LC	1	0	0	0	0	0	0	0	1	0	0	0	0	0
Platyrinchus mystaceus	bird	insectivore	LC	1	0	0	0	0	0	1	0	0	0	0	0	0	0
Ramphocelus bresilius	bird	small frugivore	LC	1	0	0	0	0	0	0	0	1	0	0	0	0	0
Rupornis magnirostris	bird	omnivore	LC	1	0	0	0	0	0	0	0	0	0	1	0	0	0
Sittasomus griseicapillus	bird	insectivore	LC	1	0	0	0	1	0	0	0	0	0	0	0	0	0
Urubitinga urubitinga	bird	carnivore	LC	1	0	0	0	0	0	1	0	0	0	0	0	0	0
Xiphocolaptes albicollis	bird	omnivore	LC	1	0	0	0	0	0	0	0	0	0	1	0	0	0
Ramphastos vitellinus	bird	large frugivore	VU	0	1	0	0	0	0	0	0	0	1	0	0	0	0

*Domestic species. **Small rodents: small mammal rodents unidentified. States Parks in CB: Carlos Botelho; SV-I: Itamambuca, SV-VG: Vargem Grande, IC. Ilha do Cardoso, INT: Intervales, IB: Ilhabela. IUCN: Red List of Threatened Species – LC: least concern, NT: near threatened, VU: vulnerable, EN: endangered. *Visits = number of visits per site, Freq=frequency, States Parks in CB: Carlos Botelho; SV-I: Itamambuca, SV-VG: Vargem Grande, IC. Ilha do Cardoso, IB: Ilhabela, INT: Intervales.

Electronic Supplemental Material 3

Defaunation index of sampling sites in the Atlantic Forest, Brazil. The index is based on the frequency of species of medium and large mammals and their body mass in a reference area (Giacomini and Galetti, 2013) and in the States Parks in CB: Carlos Botelho; SV-I: Itamambuca, SV-VG:

Taxon	Frequency (%)	CB (visits)	CB (freq.)	SV-I (visits)	SV-I (freq.)	SV-VG (visits)	SV-VG (freq.)	IC (visits)	IC (freq.)	IB (visits)	IB (freq.)	INT (visits)	INT (freq.)
Dasyprocta sp.	87.69	0	0.00	0	0.00	1	3.44	0	0.00	0	0	0	0,00
Dasypus sp.	76.15	1	3.33	1	4.16	1	3.44	1	4.16	8	40	1	4,16
Eira barbara	72.31	3	10.00	7	29.16	3	10.34	0	0.00	0	0	4	16,66
Leopardus sp.	56.92	8	26.66	11	45.83	5	17.24	4	16.66	2	10	7	29,16
Mazama sp.	69.23	8	26.66	0	0.00	0	0.00	4	16.66	0	0	6	25,00
Nasua nasua	73.08	3	10.00	2	8.33	0	0.00	0	0.00	0	0	0	0,00
Panthera onca	26.15	0	0.00	0	0.00	0	0.00	0	0.00	0	0	1	4,16
Pecari tajacu	69.23	1	3.33	4	16.66	1	3.44	0	0.00	0	0	3	12,50
Puma concolor	51.54	4	13.33	7	29.16	8	27.58	3	12.50	0	0	5	20,83
Tapirus terrestris	38.46	13	43.33	10	41.66	2	6.89	0	0.00	0	0	10	41,66
Tayassu pecari	33.08	6	20.00	14	58.33	1	3.44	14	58.33	0	0	0	0,00
**Defaunation index**			**0.31**		**0.23**		**0.73**		**0.68**		**0.93**		**0.32**

Vargem Grande, IC. Ilha do Cardoso, IB: Ilhabela, INT: Intervales.

Electronic Supplemental Material 4

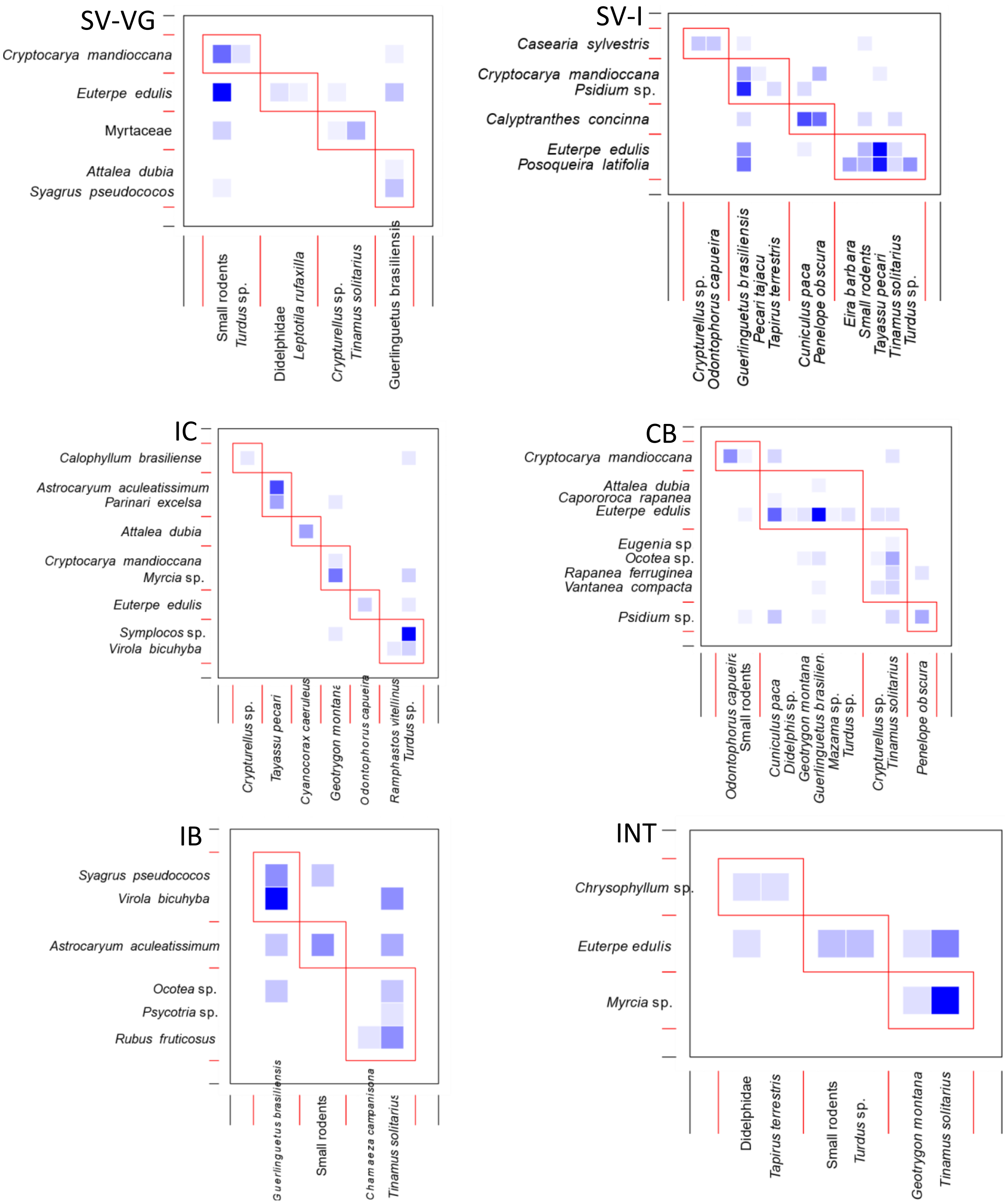

Fig 4 Modules in the plant–frugivore interaction matrix. The matrix shows the most common modules. The intensity of shades of blue represents the interaction frequency. The darker the blue the greater the number of interactions performed in the Atlantic forest, Brazil. State Parks: SV-I – Itamambuca, CB - Carlos Botelho, INT- Intervales, SV-VG -Vargem Grande, IC - Ilha do Cardoso and IB -Ilhabela.

Electronic Supplemental Material 5

Species strength of species of mammals, birds and trees in the Atlantic forest, Brazil. State Parks: SV-I – Itamambuca, CB - Carlos Botelho, INT- Intervales, SV-VG -Vargem Grande, IC - Ilha do Cardoso and IB -Ilhabela.

		Species strenght					
	Species	CB	INT	SV-I	SV-VG	IC	IB
Mammals	*Cuniculus paca*	1.76	--	0.57	--	--	--
	Didelphidae	--	0.60	--	0.08	--	--
	Didelphis aurita	0.03	--	--	--	--	--
	Eira barbara	--	--	0.13	--	--	--
	Guerlinguetus brasiliensis	1.88	--	1.84	2.04	--	2.08
	Mazama sp.	0.03	--	--	--	--	--
	Pecari tajacu	--	--	0.09	--	--	--
	Small rodents	0.16	0.20	0.46	1.98	--	0.78
	Tapirus terrestris	--	0.50	0.12	--	--	--
	Tayassu pecari	--	--	0.95	--	1.80	--
Birds	*Chamaeza campanisona*	--	--	--	--	--	0.20
	Crypturellus sp.	0.48	--	0.37	0.15	0.50	--
	Cyanocorax caeruleus	--	--	--	--	1.00	--
	Geotrygon montana	0.15	0.21	--	--	2.03	--
	Leptotila rufaxilla	--	--	--	0.04	--	--
	Odontophorus capueira	0.57	--	0.37	--	0.67	--
	Penelope obscura	0.80	--	0.69	--	--	--
	Ramphastos vitellinus	--	--	--	--	0.33	--
	Tinamus solitarius	3.09	1.29	0.21	0.55	--	2.94
	Turdus albicollis	0.05	0.20	0.15	--	--	--
	Turdus rubiventris	--	--	--	0.15	--	--
	Turdus sp.	--	--	--	--	2.67	--
Plants	*Astrocaryum aculeatissimum*	--	--	--	--	0.67	0.10
	Attalea dubia	0.04	--	--	0.10	1.00	--
	Calophyllum brasiliense	--	--	--	--	1.06	--
	Calyptranthes concinna	--	--	2.01	--	--	--
	Capororoca rapanea	0.05	--	--	--	--	--

Casearia sylvestris	--	--	2.12	--	--	--
Chrysophyllum sp.	--	1.50	--	--	--	--
Cryptocarya mandioccana	1.59	--	1.52	1.42	0.11	--
Eugenia sp.	0.05	--	--	--	--	--
Euterpe edulis	5.86	3.33	1.45	3.45	1.06	--
Myrcia sp.	--	1.17	--	--	0.78	--
Myrtaceae	--	--	--	1.60	--	--
Ocotea sp.	0.92	--	--	--	--	0.26
Parinari excelsa	--	--	--	--	0.44	--
Posoqueira latifolia	--	--	3.40	--	--	--
Psidium sp.	1.49	--	1.51	--	--	--
Psycotria sp.	--	--	--	--	--	0.07
Rapanea ferruginea	0.40	--	--	--	--	--
Rubus fruticosus	--	--	--	--	--	1.28
Syagrus pseudococos	--	--	--	0.43	--	0.57
Symplocos sp.	--	--	--	--	0.76	--
Vantanea compacta	0.59	--	--	--	--	--
Virola bicuhyba	--	--	--	--	1.12	0.81
